# Inhibition of *miR-181a* promotes midbrain neuronal growth through a Smad1/5-dependent mechanism: implications for Parkinson’s disease

**DOI:** 10.1042/NS20170181

**Published:** 2018-01-26

**Authors:** Shane V. Hegarty, Aideen M. Sullivan, Gerard W. O’Keeffe

**Affiliations:** 1 Department of Anatomy and Neuroscience and Cork Neuroscience Centre, Western Gateway Building, University College Cork (UCC), Cork, Ireland; 2 APC Microbiome Institute, UCC, Cork, Ireland; 3 INFANT Centre, Cork University Maternity Hospital and UCC, Cork, Ireland

**Keywords:** axon, dopamine, microRNA

## Abstract

Parkinson’s disease (PD) is the second most common neurodegenerative disease, and is characterized by the progressive degeneration of nigrostriatal dopaminergic (DA) neurons. Current PD treatments are symptomatic, wear off over time and do not protect against DA neuronal loss. Finding a way to re-grow midbrain DA (mDA) neurons is a promising disease-modifying therapeutic strategy for PD. However, reliable biomarkers are required to allow such growth-promoting approaches to be applied early in the disease progression. *miR-181a* has been shown to be dysregulated in PD patients, and has been identified as a potential biomarker for PD. Despite studies demonstrating the enrichment of *miR-181a* in the brain, specifically in neurites of postmitotic neurons, the role of *miR-181a* in mDA neurons remains unknown. Herein, we used cell culture models of human mDA neurons to investigate a potential role for *miR-181a* in mDA neurons. We used a bioninformatics analysis to identify that *miR-181a* targets components of the bone morphogenetic protein (BMP) signalling pathway, including the transcription factors Smad1 and Smad5, which we find are expressed by rat mDA neurons and are required for BMP-induced neurite growth. We also found that inhibition of neuronal *miR-181a*, resulted in increased Smad signalling, and induced neurite growth in SH-SY5Y cells. Finally, using embryonic rat cultures, we demonstrated that *miR-181a* inhibition induces ventral midbrain (VM) and cortical neuronal growth. These data describe a new role for *miR-181a* in mDA neurons, and provide proof of principle that *miR-181a* dysresgulation in PD may alter the activation state of signalling pathways important for neuronal growth in neurons affected in PD.

## Introduction

Parkinson’s disease (PD) is the second most common neurodegenerative disease that presents with both motor and non-motor symptoms, and occurs in 1–2% of people over the age of 65 [1]. PD is characterized by the progressive degeneration of midbrain dopaminergic (mDA) neurons, and the accumulation of neuronal inclusions of aggregated α-synuclein [2]. The progressive loss of mDA neurons results in a loss of mDA striatal innervation, leading to reduced striatal dopamine levels. This leads to progressive motor impairments that are a hallmark of the disease [3]. In recent years, it is increasingly recognized that mDA axonal degeneration may be central to the progression of the early stages of PD [4,5]. This is supported by post-mortem studies showing a modest loss of mDA axons in the striatum 1 year after PD diagnosis, with a complete loss of mDA striatal innervation in patients 4 years after diagnosis, while some mDA neuronal soma remained [6]. Therefore understanding the dysfunctional molecular pathways that cause mDA axonal degeneration, and targeting these to protect or re-grow mDA axons, may be a promising therapeutic approach for PD.

Recent evidence has implicated miRNA dysregulation in PD [7]. This suggests that understanding the role of dysregulated miRNA in mDA neurons may provide insights into the pathophysiological mechanisms of mDA axonal degeneration in PD, and provide new opportunities for mDA axonal protection or re-growth. miRNA are small non-coding RNA transcripts that post-transcriptionally inhibit the expression of specific target genes to regulate many biological processes [8]. miRNA are widely expressed in the brain, where they control the development, survival, function and plasticity of neurons [9–12]. miRNA dysregulation has been implicated in the pathophysiology of a range of neurological disorders, including PD [12,13]. One miRNA, *miR-181a*, has been shown to be up-regulated in mDA neurons in PD [13]. *miR-181a* was also identified as part of a panel of miRNAs as PD biomarkers, in which circulating *miR-181a* was lower in the serum of PD patients compared with controls [14]. This is interesting given that *miR-181a* is highly expressed in the brain [9], is enriched in post-mitotic neurons [15] and is found in the growing tips of cortical axons [16], suggesting that *miR-181a* may regulate axonal growth. However, despite the evidence for *miR-181a* dysregulation in PD, the biological processes and molecular pathways regulated by *miR-181a* in mDA neurons are unclear. Here, we investigate the hypothesis that *miR-181a* regulates the molecular pathways that control mDA axonal growth, and provide evidence that *miR-181a* inhibition may be a novel therapeutic approach for axonal regeneration in PD.

## Materials and methods

### Cell culture

SH-SY5Y cells were cultured in Dulbecco’s modified Eagle’s medium nutrient mixture F-12 (DMEM/F12; Sigma), supplemented with 10% FBS (Sigma), 100 nM l-glutamine (Sigma), 100 U/ml penicillin (Sigma), 10 µg/ml streptomycin (Sigma), in a humidified atmosphere containing 5% CO_2_ at 37°C. For primary cultures, embryonic day (E)14 or E18 embryos were obtained by laparotomy from time-mated female Sprague–Dawley rats following decapitation under isoflurane-induced terminal anaesthesia. Dissected E14 ventral mesencephalon (VM) or E18 cortex was centrifuged at 1000 x ***g*** for 5 min at room temperature. The tissue pellet was then incubated in 0.1% trypsin-Hank’s Balanced Salt Solution for 5 min (25 min for cortical tissue), at 37°C with 5% CO_2_. FBS (Sigma) was added to the tissue followed by centrifugation at 1000 x ***g*** for 5 min at 4°C. The resulting cell pellet was resuspended in 1 ml differentiation medium (DMEM/F12, 33 mM d-glucose, 1% l-glutamine, 1% FBS, supplemented with 2% B27; all Sigma), and then carefully triturated using a sterile plugged flame-polished Pasteur pipette. For immunocytochemistry and neurite growth assays, a plating density of 5 × 10^4^ cells/well of a 24-well plate was used. For Western blotting, 2 ×10^6^ cells were plated per well of a six-well plate. Primary cultures were plated on poly-d-lysine (Sigma) coated tissue culture plates. Where indicated, cells were treated daily with 10 or 200 ng/ml of growth differentiation factor (GDF) 5 (GDF5) (Biopharm GmbH), which are maximal saturating concentrations in primary and SH-SY5Y cell cultures respectively [17,41]. At these concentrations, GDF5 promotes neurite growth and activates intracellular Smad signalling to a similar order of magnitude through a common bone morphogenetic protein (BMP) receptor (BMPR)-dependent mechanism in these two cell models.

### Electroporation of cultured cells

Electroporation of cultured cells was carried out using the Neon Transfection System (Invitrogen). Dissociated cell suspensions were prepared and the required volume of cells to give 2 ×10^5^ cells per well was centrifuged at 1000 x ***g*** for 5 min at 4°C. The cell pellet was resuspended in 12 µl of the manufacturers resuspension buffer per transfection/plasmid (Invitrogen); and 0.5 µg of *GFP* plasmid; 1 µg of *Smad4* siRNA vector [17]; 100 nM of control, *Smad1* or *Smad5* siRNA (Life Technologies); or 100 nM of control miRNA or *miR-181a* inhibitor/antagomir (Life Technologies) was added to the cell suspension. Ten microlitres of the cell/plasmid mixture was then electroporated according to the manufacturer’s instructions using the following parameters (1200 V, 20 ms, 3 pulses for SH-SY5Y cells or 1100 V, 30 ms, 2 pulses for primary cultures).

### Immunocytochemistry

Immunocytochemistry was performed as previously described [17]. Briefly, cultured cells were fixed, washed, blocked and subsequently incubated in the following primary antibodies: p-Smad 1/5 (1:200; Cell Signaling) and tyrosine hydroxylase (TH) (1:200; mouse monoclonal; Millipore). Alexa Fluor 488- and/or 594-conjugated secondary antibodies (1:500; Invitrogen) reactive to the species of the primary antibodies, and the Olympus IX70 inverted microscope, were used for visualization. For densitometry analysis, the fluorescence intensity of individual cells stained for p-Smad1/5 was measured using ImageJ analysis software. The relative fluorescence intensity was calculated for individual cells after subtraction of the background.

### Analysis of neuronal complexity

Neurite length was measured as described in [18]. Neurite length was calculated using the following formula; NL = α × T × (π/2), where α is the number of times the neurite intersects the grid lines and T is the distance between the gridlines on the magnified image (taking into account the magnification factor) or was calculated using ImageJ analysis software.

### Western blotting

Western blotting was carried out as described [19]. Cultured cells were lysed in radioimmunoprecipitation assay (RIPA) buffer (50 μl RIPA per 1 × 10^6^ cells; 50 mM Tris/HCl; 150 mM NaCl, 1% Triton X-100, 1 mM EDTA, 1 mM NaF, 1 mM Na_3_VO_4_, 1 μg/ml leupeptin and 1 μg/ml pepstatin) for 1 h on ice, and insoluble debris was removed by centrifugation. Fifteen micrograms protein was run by SDS/PAGE and transferred to nitrocellulose membranes using a Mini Trans-Blot Electrophoretic Transfer Cell (Bio–Rad, CA, U.S.A.). The membranes were incubated with primary antibodies against p-Smad1/5 (1:1000), Smad1/5 (1:1000; Abcam) or β-actin (1:2000) overnight at 4°C, washed, incubated with the appropriate goat IR700/800-labelled secondary antibodies (1:10000; LICOR), washed and visualized with Odyssey (LICOR). Protein levels were normalized to total protein by densitometry using Image Studio Lite software (LICOR).

### Statistical analysis

Unpaired Student’s *t* test or one-way ANOVA with a post-hoc Tukey’s test was performed, as appropriate, to determine significant differences between groups. Results were expressed as means with S.E.M. and deemed significant when *P*<0.05.

## Results

### *In silico* analysis implicates *miR-181a* as a regulator of BMP/GDF signalling

We first sought to gain insight into the biological processes that may be altered by elevated *miR-181a* expression in the mDA neurons in PD [13]. To do this, we used miRecords to create a list of putative *miR-181a* mRNA targets predicted by at least four prediction programs [20]. The search strategy included a search of ‘Validated’ and ‘Predicted’ targets of *miR-181a* in miRecords. We then compiled a list of 881 predicted mRNA targets that unambiguously mapped to unique Entrez Gene IDs, and performed a number of enrichment analyses using the WebGestalt platform [21] to identify functional categories and pathways overrepresented in this gene list. Gene ontology (GO) enrichment analysis revealed a statistically significant overrepresentation of genes associated with the GO categories: ‘nervous system development’ (GO:0007399) and ‘neuron projection’ (GO:0043005) (Table 1). We next sought to determine whether there was an overrepresentation of genes associated with specific KEGG pathways in the list of *miR-181a* target genes. There was a statistically significant overrepresentation of genes associated with four KEGG pathways that have been shown to affect mDA neuronal growth. These included the ‘neurotrophin signalling pathway’ [22], ‘transforming growth factor-β (TGF-β) signalling pathway’ [23], ‘MAPK signalling pathway’ [24] and ‘Wnt signalling pathway’ [25] (Table 1). Given that TGF-β superfamily members are potent neurotrophic factors for mDA neurons, and promote the neurite growth of these neurons [26,27], we examined genes associated with the ‘TGF-β signalling pathway’. We found that these target genes included the receptors BMP receptortype II (BMPR2; ENSG00000204217) and activin A receptor type IIA (ACVR2A; ENSG00000121989). Both receptors are known to bind and mediate signalling by bone morphogenetic proteins (BMPs), and the related GDFs, which are major subgroups of the TGF-β superfamily [23,28]. Given that this suggested a role for *miR-181a* in regulating BMP/GDF signalling, we next used the DIANA-TarBase v7.0 platform to search for experimentally validated targets of *miR-181a* that are known to play functional roles in BMP/GDF signaling [29]. In addition to BMPR2 and ACVR2A, we found a number of experimentally validated targets of *miR-181a* that play key roles in BMP/GDF signalling including: SMAD1 [30] and SMAD5 [31] transcription factors—the downstream effector proteins of the canonical BMP/GDF signalling pathway [32,33]; and Zinc finger E-box-binding homoeobox 2 (Zeb2) [34]—a key transcriptional regulator of BMP-Smad signalling [35,36] that controls neurite growth in mDA neurons [37] (Table 2). We therefore hypothesized that *miR-181a* may play a functional role in modulating BMP-Smad signalling to control neurite growth in mDA neurons.

**Table 1:** GO categories and KEGG pathways represented in the list of hsa-*miR-181a* predicted target genes relative to the background gene population

ID	Category name	Observed genes	Expected genes	Enrichment ratio	Adjusted *P* value
**GO term**					
GO:0007399	Nervous system development	161	90.27	1.78	1.46 × 10^−10^
GO:0043005	Neuron projection	60	31.57	1.90	8.39 × 10^−05^
**KEGG**					
04722	Neurotrophin signalling pathway	18	2.59	6.94	1.82 × 10^−08^
04350	TGF-β signaling pathway	10	1.72	5.83	5.36 × 10^−05^
04010	MAPK signalling pathway	24	5.47	4.38	5.48 × 10^−08^
04310	Wnt signalling pathway	13	3.06	4.24	7.64 × 10^−05^

**Table 2:** Experimentally validated targets of hsa-*miR-181a* with roles in BMP/GDF signalling

Ensembl gene ID	Symbol	Description	Role in BMP/GDF signalling
ENSG00000204217	BMPR2	Bone MPR2	Ligand-binding receptor
ENSG00000114739	ACVR2A	ACVR2A	Ligand-binding receptor
ENSG00000170365	SMAD1	SMAD family member 1	Signal transducer and transcriptional modulator
ENSG00000113658	SMAD5	SMAD family member 5	Signal transducer and transcriptional modulator
ENSG00000113658	ZEB2	Zeb2	Transcriptional modulator
ENSG00000168610	STAT3	Signal transducer and activator of transcription 3	Transcriptional modulator

### The *miR-181a* target genes Smad1/5 are required for GDF5-promoted neurite growth in primary cultures of the rat midbrain

**Figure 1:**
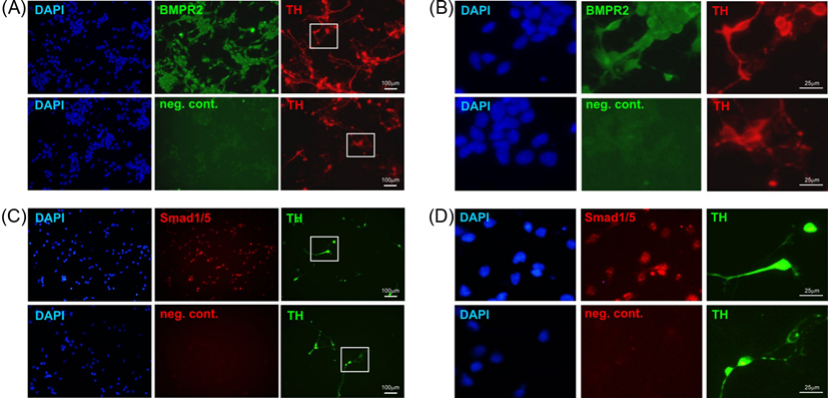
Expression of *miR-181a* target genes in primary cultures of the E14 rat VM Representative photomicrographs of TH-immunostained E14 rat VM DA neurons immunostained for BMPR2 (**A,B**) or Smad1/5 (**C,D**). Scale bar as indicated.

**Figure 2:**
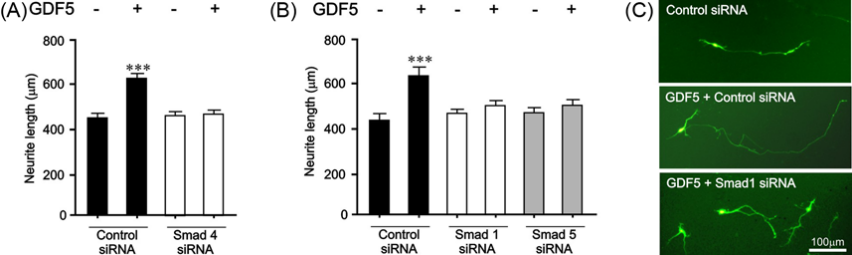
Smad1 and Smad5 signalling is important for GDF5-induced neurite growth (**A,B**) Neurite length of neurons in E14 rat VM cultures transfected with control siRNA, *Smad4* siRNA or *Smad1* siRNA or *Smad5* siRNA and a GFP-expressing plasmid before being cultured with or without 10 ng/ml GDF5 for 72 h (***P<0.001 compared with control; ANOVA with post-hoc Tukey’s test; 40 cells for each group per experiment; number of repetitions (*n*)=3 experiments). All data are presented as mean ± S.E.M. (**C**) Representative photomicrographs of GFP+ neurons in cultures of E14 rat VM. Scale bar as indicated.

To begin to test this hypothesis, we first used primary cultures of the E14 rat ventral midbrain (VM), which are an experimental tractable model of mDA neurons that are widely used to study the molecular mechanisms regulating mDA neurite growth [18]. We used immunocytochemistry to examine whether the validated *miR-181a* target genes were expressed on mDA neurons. Triple-labelled preparations in which mDA neurons were identified by TH staining revealed that BMPR2 was expressed on, but not limited to, mDA neurons in cultures of the E14 rat VM (Figure 1A,B). Moreover, we also found that the Smad1 and Smad5 transcription factors were expressed in a similar manner to BMPR2 in these cultures (Figure 1C,D). Having confirmed that the *miR-181a* target genes were expressed in these cultures, we next examined the functional importance of the BMP/GDF receptor-regulated Smads, Smad1 and Smad5, for the neurite growth induced by BMP/GDF ligands. Given that BMPR2 and ACVR2A were predicted and validated targets of *miR-181a* (Table 2), the cells were treated with GDF5 (also known as BMP14) as a positive control, because GDF5 is known to bind BMPR2 or ACVR2A and activate Smad signaling [28]. Treatment with 10 ng/ml of GDF5 for 72 h led to significant increases in neurite length (*P*<0.001) (Figure 2A,C). To examine the functional role of Smad1 and Smad5 in GDF5-induced neurite length, primary cultures were transfected by microporation with siRNAs targeting the co-Smad4 (required for nuclear translocation of Smad1/5 transcriptional complex [33]) or the Smad1 and Smad5 effectors proteins, which are known targets of *miR-181a* (Table 2). We found that siRNAs targeting Smad 4 (Figure 2A) or Smad1 and Smad5 (Figure 2B,C) prevented GDF5-promoted neurite growth in E14 rat VM cultures. These data show that *miR-181a* targets genes are expressed in, but not limited to mDA neurons, which play a crucial role in neurite growth induced by a ligand of the BMP/GDF subfamily.

### *miR-181a* inhibition promotes Smad1/5 phosphorylation and neurite growth in SH-SY5Y cells

**Figure 3:**
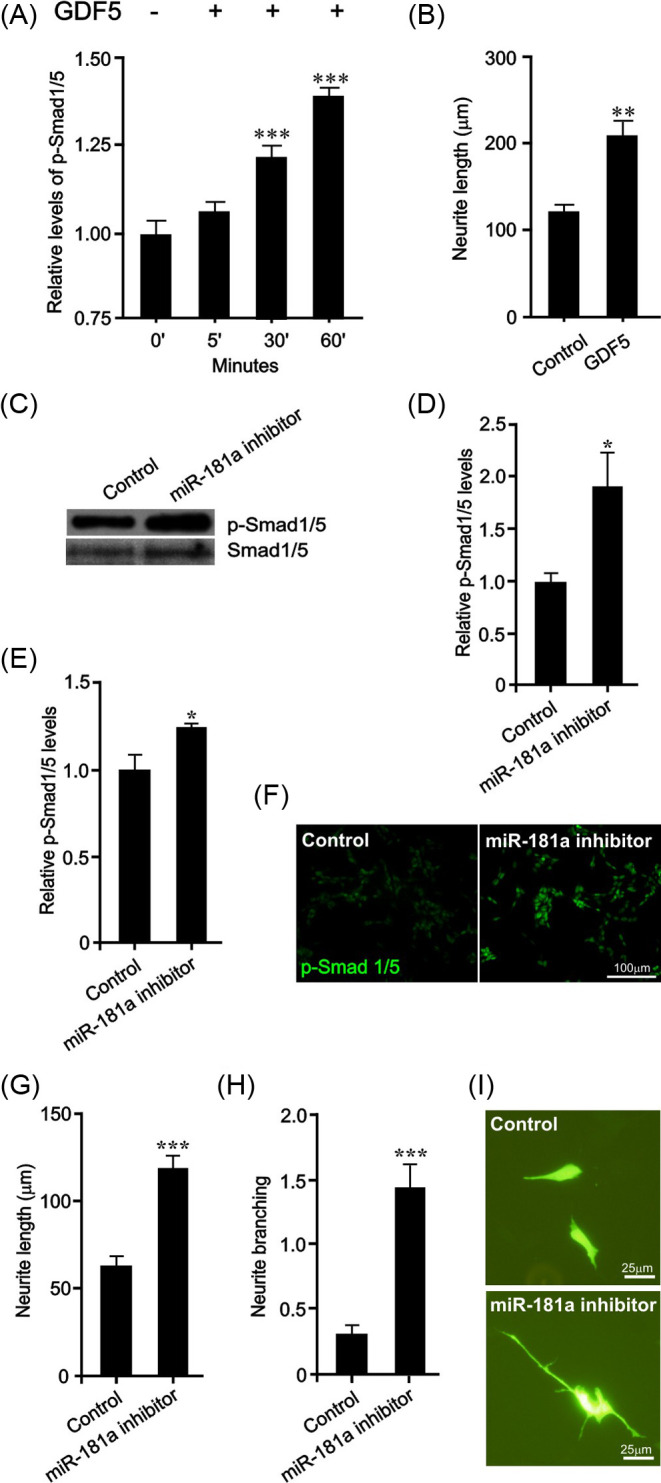
*miR-181a* inhibition promotes Smad1/5 phosphorylation and neurite growth in SH-SY5Y cells (**A**) Densitometric analysis of p-Smad1/5 in SH-SY5Y cells treated with 200 ng/ml of GDF5 for 0 (control), 5, 30 and 60 min. (**B**) Neurite length of GDF5-treated (200 ng/ml daily for 72 h) SH-SY5Y cells at 72 h. (**C–F**) p-Smad1/5 levels as determined by (**C, D**) Western blots or (**E, F**) immunocytochemistry in SH-SY5Y cells 24 h after transfection with control miRNA or *miR-181a* inhibitor. Total Smad1/5 was used as a loading control. (**G**) Neurite length, (**H**) neurite branching and (**I**) representative photomicrographs of *miR-181a* inhibitor or control miRNA transfected SH-SY5Y cells at 72 h (*P<0.05, **P<0.01, ***P<0.001 compared with control; *t* test; 40 cells for each group per experiment; *n*=3 experiments). All data are presented as mean ± S.E.M. Scale bar as indicated.

Having established the importance of the *miR-181a* target genes, Smad1 and Smad5, in BMP/GDF ligand-induced neurite growth, we next determined whether *miR-181a*-modulated Smad phosphorylation/activation and neurite growth in SH-SY5Y cells, which are a widely used cell line model of human mDA neurons which also express the *miR-181a* targets, BMPR2 and Smad1/5 [18,38]. This cell line permits higher transfection efficiencies than primary neurons which facilitated molecular and biochemical analyses [18]. Firstly, SH-SY5Y cells where treated with 200 ng/ml of GDF5 for various time points for up to 60 min to examine Smad1/5 phosphorylation, or for 72 h to examine neurite growth. GDF5 led to a significant increase in p-Smad1/5 at 30 min (*P*<0.001) and 60 min (*P*<0.001) (Figure 3A), and a significant increase in neurite growth at 72 h (*P*<0.01) (Figure 3B). We next sought to establish the functional role of endogenous *miR-181a*. To do this, SH-SY5Y cells were transfected with a *miR-181a* inhibitor or a scrambled control, and then cultured for 24 h before p-Smad1/5 levels were examined by Western blotting (Figure 3C,D) and immunocytochemistry (Figure 3E,F) and quantified by densitometry. We found that similar to treatment with GDF5, transfection with the *miR-181a* inhibitor led to a significant increase in the levels of p-Smad1/5 (Figure 3C-F). To determine whether the increase in p-Smad1/5 levels were paralleled by an increase in neurite growth, SH-SY5Y cells were transfected with an *miR-181a* inhibitor or a scrambled control and cultured for 72 h before neurite growth was examined using a modified line intercept method as previously described [18]. Similar to GDF5 treatment, we found that transfection with the *miR-181a* inhibitor led to significant increases in neurite length (Figure 3G,I) (*P*<0.001) and neurite branching (Figure 3H,I) (*P*<0.001) compared with the controls. These data show that *miR-181a* regulates BMP-Smad signalling and neurite growth in SH-SY5Y cells.

### Smad1 and Smad5 are required for neurite growth induced by *miR-181a* inhibition

**Figure 4:**
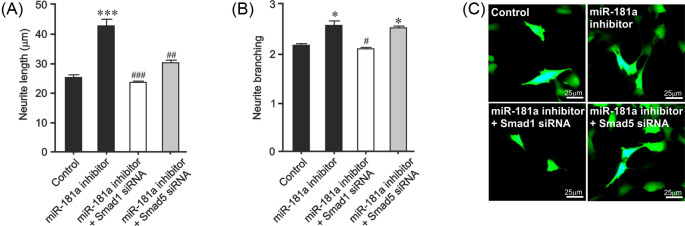
Smad1 and Smad5 are important for neurite growth induced by *miR-181a* inhibition (**A**) Neurite length and (**B**) neurite branching of SH-SY5Y cells transfected with control siRNA or *Smad1* siRNA or *Smad5* siRNA and a GFP-expressing plasmid together with a control or *miR-181a* antagomir for 72 h (*P<0.05, ***P<0.001 compared with control) (^#^*P*<0.05, ^##^*P*<0.01, ^###^*P*<0.001 ANOVA with post-hoc Tukey’s test; 40 cells for each group per experiment; *n*=3 experiments). All data are presented as mean ± S.E.M. (**C**) Representative photomicrographs of GFP+ neurons in cultures of E14 rat VM. Scale bar as indicated.

Given that Smad 1/5 are required for neurite growth induced by BMP ligands (Figure 2), we next sought to test the hypothesis that the *miR-181a* inhibitor mediated Smad1/5 activation is responsible for the increase in neurite growth following *miR-181a* inhibition. To do this, SH-SY5Y cells were transfected with siRNAs targeting Smad1 or Smad5 or with a scrambled siRNA, with or without the *miR-181a* antagomir or a control. We found that *miR-181a* inhibition led to a significant increase in neurite length (*P*<0.001) (Figure 4A,C). Smad1 siRNA completely prevented the increases in neurite length induced by *miR-181a* inhibition (Figure 4A,C). Similarly, Smad5 siRNA partially prevented the neurite growth induced by miR-181a inhibition. We also found that *miR-181a* inhibition lead to a significant increase in neurite branching (*P*<0.05), while was completely prevented by Smad1, but not Smad5 siRNA (Figure 4B,C). These data show that *miR-181a* inhibition promotes neurite growth through a Smad1- and, to a lesser extent, Smad5-dependent mechanism.

### *miR-181a* inhibition promotes neurite growth in BMP-responsive neuronal populations

**Figure 5:**
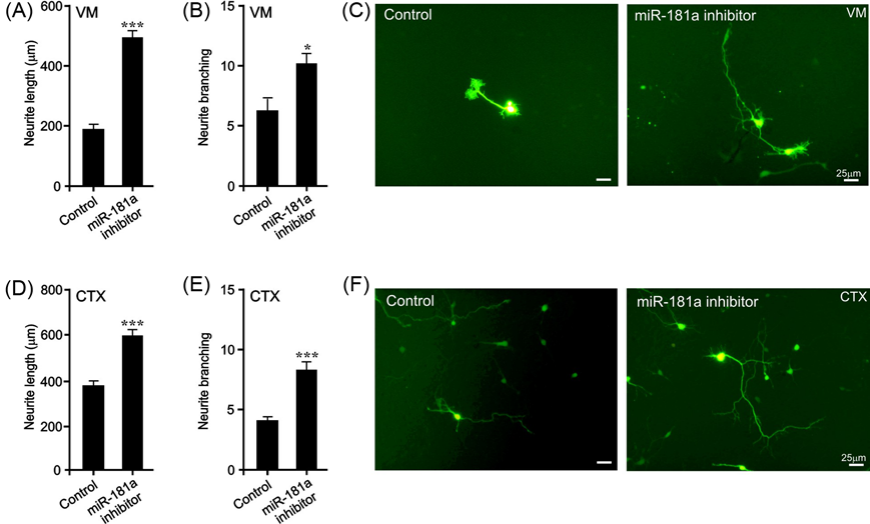
*miR-181a* inhibition induces neurite growth in BMP-responsive primary neurons Neurite length (**A**), neurite branching (**B**) and photomicrographs (**C**) of *miR-181a* inhibitor- or control miRNA-transfected neurons in E14 rat VM cultures at 72 h. Neurite length (**D**), neurite branching (**E**) and photomicrographs (**F**) of *miR-181a* inhibitor or control miRNA-transfected neurons in E18 rat cortical (CTX) cultures at 48 h. (*P<0.05, ***P<0.001 compared with control; *t*test; 40 cells for each group per experiment; *n*=3 experiments). All data are presented as mean ± S.E.M. Scale bar as indicated.

We next sought to determine whether transfection with the *miR-181a* inhibitor could also promote neurite growth in E14 VM neurons, a widely used model of mDA neurons [18]. To do this, E14 VM neurons were microporated with the *miR-181a* inhibitor or a scrambled control, and a GFP-expression plasmid to identify the transfected cells. Individual GFP-expressing neurons were imaged 72 h later and neurite length and branching was quantified using a modified line intercept method [18]. We found that *miR-181a* inhibition led to a significant increase in neurite length (*P*<0.01) (Figure 5A,C) and neurite branching (*P*<0.01) (Figure 5B,C). To confirm these findings, we also examined the effect of *miR-181a* inhibition in a second BMP-responsive neuronal population, cortical neurons [39]. Similar to the findings in E14 VM neurons, we found that *miR-181a* inhibition also led to significant increase in neurite length (*P*<0.01) (Figure 5D,F) and neurite branching (*P*<0.01) (Figure 5E,F) at 48 h in E18 rat cortical neurons. These data show *miR-181a* inhibition promotes neurite growth and branching in midbrain and cortical neurons.

## Discussion

At present, there is an urgent clinical need to extend our understanding of PD pathology, to develop novel disease-modifying therapies for PD, and to identify reliable PD biomarkers to allow for earlier interventions. *miR-181a* has been shown to be dysregulated in PD patients, suggesting that it may contribute to the underlying pathology, while it has also been proposed as a potential disease biomarker [13,14]. Despite studies demonstrating the enrichment of *miR-181a* in the brain, but more specifically in the neurites of post-mitotic neurons [9,15,16,40], the role of *miR-181a* in mDA neurons remained unknown. Herein, we used cell culture models of human mDA neurons [18] to investigate the function of *miR-181a*.

Firstly, we carried out an *in silico* analysis that identified members of the TGF-β superfamily, including BMPR2 and ACVR2A, as targets of *miR-181a*. We focused on this pathway as a number of TGF-β superfamily members are potent neurotrophic factors for mDA neurons that promote neurite growth and arborization [26,27]. We then found that the transcription factors Smad1, Smad5 and Zeb2 were also experimentally validated targets of *miR-181a* [30,31,34], which are key downstream regulators of BMP/GDF signalling [32,33,35,36]. Therefore we hypothesized that *miR-181a* may modulate BMP-Smad signalling to control neurite growth in mDA neurons. After demonstrating that cultured mDA neurons express BMPR2 and Smad1/5, we found that Smad1 and Smad5 are required for the neurite growth-promoting effects of GDF5 (BMP14), which binds to BMPR2 and ACVR2A and activates Smad signalling [28]. Thus, *miR-181a* target genes are expressed by mDA neurons, and play crucial roles in BMP/GDF-induced neurite growth.

The present study and others have demonstrated that the *miR-181a* target genes, Smad1, Smad5 and Zeb2 are important regulators of BMP/GDF-induced mDA neuronal growth [37,41]. To investigate whether *miR-181a* plays a role in these effects, we transfected SH-SY5Y cells, which are a well-established model of human mDA neurons [18,38], with an miR-181a inhibitor/antagomir. Inhibition of miR-181a resulted in induction of Smad signalling and led to the promotion of neurite growth in these cells, in a similar fashion to that induced by the physiological ligand, GDF5, which was used as a positive control. Given that BMPR2, ACVR2A, Smad1 and Smad5 are targets of *miR-181a*, it is logical that *miR-181a* inhibition promoted Smad signalling. This finding is also supported by a previous report showing that *miR-181a* inhibition can induce Smad signalling in HepG2 cells [42]. Interestingly, *miR-181a* expression has been shown to be up-regulated by TGF-β and BMP-Smad signalling [43,44], which suggests that miR-181a may participate in a negative feedback loop to limit BMP-Smad-induced mDA neuronal growth. Moreover, *miR-181a* expression has also been shown to be up-regulated by dopamine [40], suggesting that DA neurotransmission may limit neurite growth through a feedback mechanism also.

We next sought to determine whether transfection with the *miR-181a* inhibitor could also promote neurite growth in primary cultures of E14 VM neurons, which are widely used models of mDA neurons [18]. Inhibition of miR-181a induced neurite growth in these cells also. Taken together, these data indicate that *miR-181a* may play a role in regulating neurotrophic BMP-Smad signaling and neurite growth in mDA neurons. In support of this, *miR-181a* has been shown to repress neurite growth in hippocampal neurons [45], in which *miR-181a* is enriched at synapses [40]. It is also interesting to note that GDF5-Smad signaling has been shown to promote neurite growth in hippocampal neurons [46]. Moreover, inhibition or down-regulation of *miR-181a* has been shown to be neuroprotective in the rodent forebrain *in vivo* [47,48], which is in line with our findings regarding the neuritogenic effects of *miR-181a* inhibition.

In terms of pathogenic mechanisms of PD, given that *miR-181a* is selectively up-regulated in nigrostriatal DA neurons of the PD brain [13], our current findings suggest that this up-regulation in *miR-181a* may contribute to the ongoing mDA axonal degeneration by inhibiting BMP-Smad signalling *in vivo*. This hypothesis would also be consistent with the finding that TGF-β and BMP ligands have potent neuroprotective effects in mDA neurons *in vivo* [26,27]. Finally, *miR-181a* was recently shown to induce neuronal differentiation and a DA phenotype in human long-term, self-renewing, neuroepithelial-like stem cells [49], which supports the potential role for *miR-181a* in regulating development of mDA neurons. While this may seem counter-intuitive, it is important to distinguish between the specification of mDA neurons and their later morphological development. For example, the expression of BMP-Smad signaling pathway components is low during differentiation of mDA neurons from their VM neural precursors *in vivo* (E10–E12), yet after this period, these subsequently increase in expression and play a functional role in mDA neuronal outgrowth (E1–E18) [37]. Perhaps *miR-181a* plays a temporal- and developmental stage-dependent regulatory role during this neurogenic process, in a similar fashion to that of its target Zeb2 [37].

These data indicate that *miR-181a* regulates neurite growth in BMP-responsive neuronal populations, so we investigated the effects of the *miR-181a* inhibitor in cortical neurons, in which BMPR2 mediates BMP-dependent dendritogenesis [39]. Similar to the findings in E14 VM neurons, we found that *miR-181a* inhibition also led to a significant increase in neurite growth in cortical neurons. As aforementioned, *miR-181a* has been shown to be uniquely enriched in cortical axons, being one of only two miRNAs localized as distinct granules in distal axons and growth cones [16]. Interestingly, Zeb2 also promotes growth of cortical neurons *in vitro* and *in vivo* [50]. In the future, it would be worthwhile to disentangle any potential cross-talk among BMPR2, Zeb2 and *miR-181a* in cortical neuronal growth. Moreover, this promotion of growth in both cortical and mDA neurons is interesting as there is increasing recognition of the multisystemic nature of PD. It is postulated that the underlying neuropathological processes, that is, aggregation of α-synuclein and the formation of Lewy bodies, may be initiated outside the CNS, and spread to the brainstem via autonomic projections (for recent reviews, see [51,52]). From the substantia nigra, Lewy pathology can spread via neuronal connections to many areas of the brain, resulting in the onset of multiple non-motor as well as motor symptoms (for review, see [53]). Disorders of cognition, dementia and psychosis, as well as dysexecutive syndrome, are common in PD patients (for reviews, see [54,55]). These symptoms are thought to reflect pathology in cortical neurons and dysfunction of corticostriatal circuits. In fact, there is some evidence for cognitive decline preceding motor symptoms [56], meaning that studies investigating the neuropathological processes involved in cortical dyfunctionin PD, as well as the development of therapies to prevent these processes, are critically important.

In summary, the present study has shown that *miR-181a* is a novel regulator of BMP-Smad signalling and neurite growth, and proposes that targeted inhibition of *miR-181a*, given that it is up-regulated in the SN in PD, may be a novel strategy to promote growth of mDA and cortical neurons in regenerative therapies for this disorder.

## Summary

Axonal degeneration of dopamine-producing neurons is central to the progression of the early stages of PD. Identifying and targeting the molecules that cause axonal degeneration is needed to identify much needed new therapies. Here, we focus on a miRNA, known as *miR-181a*, whose expression has been found to be altered in people with PD. We report that targeting *miR-181a* regulates axonal growth, and that *miR-181a* inhibition may be a novel therapeutic approach for axonal regeneration of neurons affected by PD.

## Abbreviations

ACVR2A: activin A receptor type IIA
BMP: bone morphogenetic protein
BMPR: BMP receptor, type II
CNS: Central nervous system
DA: dopaminergic
DMEM/F12: Dulbecco’s modified Eagle’s medium nutrient mixture F-12
E: embryonic day
GDF: growth differentiation factor
GO: gene ontology
KEGG: Kyoto encylopedia of genes and genomes
MAPK: Mitogen-activated protein kinase
mDA: midbrain dopaminergic
PD: Parkinson’s disease
RIPA: radioimmunoprecipitation assay
SN: Substantia nigra
TGF-β: transforming growth factor-β
TH: tyrosine hydroxylase
VM: ventral midbrain/mesencephalon
Zeb2: Zinc finger E-box-binding homoeobox 2
